# Environment-Driven Changes in the Functional Traits of Milk Thistle [*Silybum marianum* (L). Gaertn.] Along an Altitudinal Gradient in the Semi-Arid Environment: Perspective on Future Plant Invasion

**DOI:** 10.3389/fpls.2022.897678

**Published:** 2022-06-27

**Authors:** Nasrullah Khan, Rafi Ullah, Saud S. Alamri, Yasmeen A. Alwasel, Abdulrahman AL-Hashimi, Mostafa A. Abdel-Maksoud, Mohammad K. Okla, Hamada AbdElgawad

**Affiliations:** ^1^Department of Botany, University of Malakand, Chakdara, Pakistan; ^2^Department of Botany and Microbiology, College of Science, King Saud University, Riyadh, Saudi Arabia; ^3^Laboratory for Molecular Plant Physiology and Biotechnology, Department of Biology, University of Antwerp, Antwerp, Belgium

**Keywords:** plants functional traits, elevation gradient, diversity indices, importance value index, *Silybum marianum*

## Abstract

The elevation is an important gradient across which the environmental variables and plant traits vary and is considered as a barrier to the recent global problem of plant invasion. However, certain invasive plants show plasticity traits to adapt and cope with the changes across the elevation. *Silybum marianum* (*S. marianum*) is one such invasive species widely spread in Khyber Pakhtunkhwa, Pakistan. Therefore, this study investigates the traits plasticity and invasive behaviors of this plant species across the elevation gradient. Plant functional traits (PFTs) and environmental variables were recorded in forty different low, middle, and high elevation sites. The plant shows a decrease in plant functional traits, i.e., above-ground plant height/plant, leaf length/leaf, leaf width/leaf, leaf dry weight/plant, vegetative dry weight/plant, and number of capitula/plant having the significance of *p* < 0.05. In contrast, the dry reproductive weight does not change significantly with elevation, while the root length increases across the elevation. The soil and environmental variables such as organic matter, lime percentage, and latitude significantly affected the PFTs. The importance value index of the species was also related to elevation and diversity indices, i.e., species richness, Shannon–Wiener diversity index, and evenness index, indicating that the invasion has strong effects on diversity. This study concludes that *S. marianum* has traits plasticity across the elevation and affects community diversity. Further investigation is required to understand the invasion and diversity parameters in a better way.

## Introduction

In the last several decades, transportation, tourism, business, and technological advancements have taken place as more people have moved to lowland urban areas (Fan et al., 2017). An increasing human population, coupled with the introduction and invasion of non-native species, puts natural ecosystems under increasing stress (Hulme, 2009). For commercial and non-commercial purposes, exotic plants are often transported or imported, resulting in significant geographic expansion (Brundu and Richardson, 2016). Rejm’anek and Richardson (2013) report that 434 tree species and 317 shrub species have been identified as invasive around the globe, with the majority of these species being transported from one region to another region for horticulture and urban forestry. Exotic trees are increasingly recognized as having significant invasive tendencies because of advances in invasion study (Castro-Díez et al., 2019). The importance of exotic plants was initially grown for horticultural or silvicultural objectives, but since their escaped cultivation and become naturalized in wild areas, it becomes a major problem (Catford et al., 2012).

The capacity of a plant to gather, utilize, and preserve resources is reflected in a plant’s functional traits (PFTs) (Reich et al., 2003; Violle et al., 2007; Faucon et al., 2017). Characteristics assessment is associated with plants’ potential to adapt to environmental variations and might reveal their ecological strategies and reactions to environmental variations. Besides that, plant biomass indicates plant life cycle, development, and function (Hecht et al., 2019). Environment-related variables impact the distribution of plant biomass in different organs and are intimately linked to the phenotypic traits of plants (Poorter et al., 2012; Pallas et al., 2016). It is, thus, possible that plants’ phenotypic features might help to explain the greater use of biomass (Mensah et al., 2016; Yin et al., 2019). Maintaining requires physiological activities and achieving normal development requires a delicate balancing act between stem and root biomass allocation (Shipley and Meziane, 2002; Mensah et al., 2016). Biomass allocation to plant organs that get the most limited resources is a possible way to minimize a plant’s stress under optimum allocation theory (OPT) (Mccarthy and Enquist, 2007; Freschet et al., 2018). Plant root morphology and biomass allocation have a few studies, but further study is needed.

Plant persistence, competitiveness, or resource retention varies with environment (Liu et al., 2017). It is also possible for ecosystems to use these characteristics in the face of environmental modification (Lavorel et al., 2007; Baxendale et al., 2014). According to earlier studies, the composition and structure of plant communities differ considerably with elevation (Sukopp, 2004; Croci et al., 2008; Vallet et al., 2010; McDonnell and Hahs, 2015). Interspecies characteristics along gradients can analyze how pattern of distribution change (Olden and Rooney, 2006). Studies have shown that urban stress causes plants to grow taller, but short-stature species are more vulnerable (Knapp et al., 2008; Duncan et al., 2011). Even though short-lived species (SLS) is expected to be higher in urban (low elevation) than non-urban forests (higher elevation), plant dry matter content in higher elevation will be lower than in low elevation (Diaz et al., 2010; Williams et al., 2015). Functional characteristics of plants, for example, can be used to determine how different plant species deal with environmental challenges (Palma et al., 2016).

Phenotypic plasticity refers to the ability of a particular phenotype to manifest itself under various environmental conditions (Forsman, 2015). Plasticity is the basis for a species’ survival in new adverse environmental variables (Hendry, 2016). The study of plasticity is crucial for comprehending adaptive reactions of plant in short-term life cycle (Forsman, 2015). Plant functional characteristics (PFTs) with climate change response flexibility play an important role in establishing an invasive species in non-native habitats (Yang et al., 2015). Plasticity has been reported in morphological features such as plant height, internode length, and shoot numbers. Still, the current trends include biomass allocation, relative growth, and rate of assimilations (Ullah et al., 2021). Invasive plants use morphological and physiological changes to adapt to shifting environmental conditions (Khan et al., 2020). The biomass allocation and reproductive time variables have been demonstrated to be important predictors of plant fitness (Claridge and Franklin, 2002).

*Silybum marianum* (*S. marianum*) (L.) Gaertn., commonly known as milk thistle, is an annual herb species (Roche, 1991) belonging to the Asteraceae family. The plant has long and thick leaves ranging in height from 200 to 250 cm and is mostly indigenous to temperate regions of the world (Parsons, 1973; Sindel, 1991). The plant is an herbal supplement and is extensively used to treat liver and biliary disorders (Young et al., 1978); therefore, it is grown for trade in some Asian, African, and South American countries to treat a variety of diseases (Groves and Kaye, 1989). The plant also has certain poisons like nitrates that accumulate, causing many harmful effects on the domestic animals that ingest it as a forage plant (Sindel, 1991). Moreover, the plant has extensively affected the winter crop species and has massive cover species growing in rosettes across the fields and roadside areas (Ghavami and Ramin, 2007). This study aims to analyze the variation of *S. marianum* plant functional traits (PFTs) along an altitudinal gradient. Environmental variables may be linked with resource conservation and acquisition, influencing the PFTs (morphological and biomass) along the altitudinal gradient. In addition, the importance value index (IVI) of the species was hypothesized to show negative relation with the diversity indices contributing to communities’ homogenization and varies across the altitude.

## Materials and Methods

### Sampling Area

**FIGURE 1:**
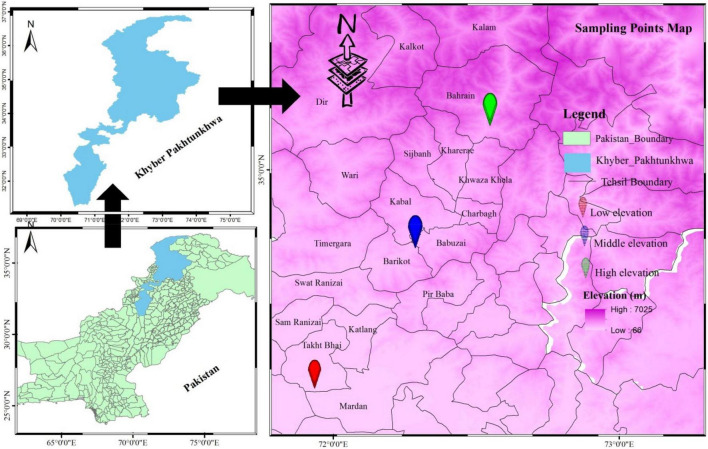
Sampling area map revealing sampling points selected for phytosociological and traits analysis (morphological and biomass).

One of Pakistan’s five administrative provinces, Khyber Pakhtunkhwa (KP) is situated in the country’s northwestern part. Azad Jammu and Kashmir (AJK) lies to the north, Afghanistan and the Federally Administered Tribal Areas (FATA) now call the merge districts to the west, Punjab to the east, and Balochistan to the south (Figure 1; Khan, 2015). The Himalayan, Hindukush, and Karakorum mountains are located in the province’s northern and eastern borders. With an altitude of 327 m in Peshawar, the province’s terrain goes from plains to mountains in the Hindukush range (Rahman and Dawood, 2016).

The temperature varies depending on the region’s altitudes, which may be found from the south to the north and northwest. There is a notable difference in temperature between the north and the south of the highlands (Ali et al., 2018). June is the hottest month, with an average high temperature of 34.96°C and a minimum temperature of 19.10°C. January is the coldest month of the year, having average low and high temperatures of 0.67 ± 0.97 and 13.72 ± 1.39°C, respectively, demonstrating harsher winter (Rahman and Khan, 2013). According to a study by Ali et al. (2018), the mean yearly precipitation varies between 384 and 639 mm, while the relative humidity ranges from 54.81 ± 2.18 to 77.35 ± 3.12% (Ali and Ali, 2018). These climatic factors are important in determining vegetation structure and composition in an area (Deo and Şahin, 2015).

### Sampling Procedure

**FIGURE 2:**
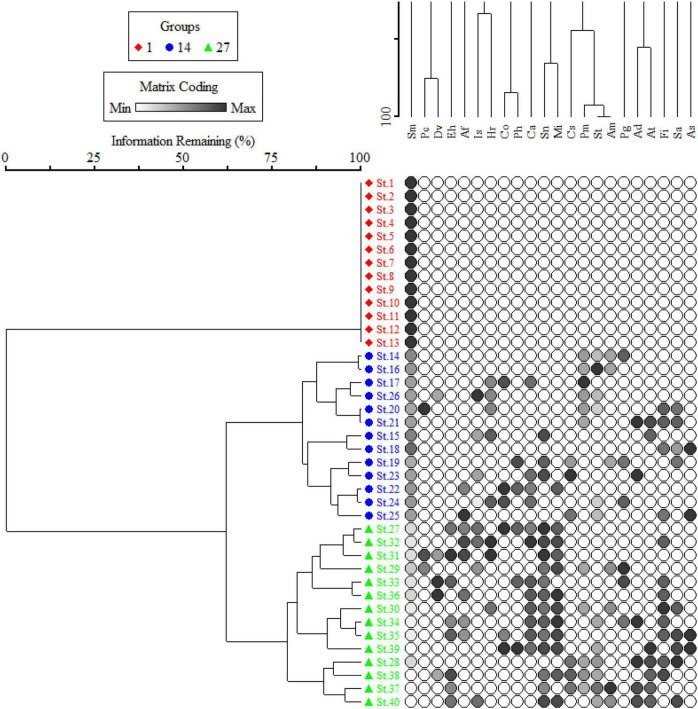
Cluster dendrogram separating 40 stands of *Silybum marianum* (*S. marianum*) across the invasion intensity.

*Silybum marianum* healthy and mature individuals were collected from 40 different sites (Figure 2), with an average of 85 plants per site (8 plants collected from the center of a 10 m × 10 m quadrat; fully invaded, severely invaded, moderately invaded, and partially invaded) and transported to the laboratory in wet towels. The plant species is mostly observed on the road, since airborne propagules spread it and, therefore, a total of 40 *S. marianum*-dominated locations were tagged for collection of phytosociological data. A 50 m × 50 m plot was established 50 m from the road and the vegetation was assessed using the quadrat method following a study by Bellhouse (2005). Each plot was splitted into three 10 m × 10 m plots, with three 5 m × 5 m subplots for documenting vegetation. The sampling sites were ruderal areas, mostly roadside, cultivated fields, and rural areas of the subtropic and dry temperate zones consisting of plain valleys, submountainous valleys, and high dry mountains zones of the region. The sites were divided into the three distinct groups based on elevation, i.e., group I includes sites located at low elevation (340.15 ± 4.84 m above sea level), group II includes sites located at intermediate elevation (859.38 ± 13.75 m above sea level), and group III includes sites located at high elevation (1548.64 ± 35.37 m above sea level). This classification correspondence with the importance value index (IVI) of *S. marianum*, where fully invaded sites were at low elevation, having 100% IVI (group I), moderate elevation sites were severely invaded, having IVI > 60% (group II), and higher elevation sites were moderate to partially invaded, having IVI > 30% (group III). A two-way cluster dendrogram further confirms the classification based on the species IVI matrix (Figure 2).

The number of capitula/plant (NC/P) and number of seeds/plant (NS/P) produced were tallied and counted manually. Following a study by Perez-Harguindeguy et al. (2016), 11 vegetative parameters were evaluated to investigate the vegetative functional traits variations and plasticity, which include root length/plant (RL/P) (cm), dry weight root/plant (DWR/P) (g), aboveground plant height/plant (AGPH/P) (cm), number of leaves/plant (NL/P), leaf diameter/leaf (LW/L) (cm), leaf length/leaf (LL/L) (cm), dry weight leaves/plant (DWLs/P) (g), dry weight stem/plant (DWS/P) (g), and crown cover/plant (CC/P) (cm). The dry weight of the plant organs was determined by oven drying the functional parts (root, stem, leaves, capitula, and seeds) at 60°C for 72 h according to the procedure adopted by Sharma et al. (2017) using an electronic balance of precision 0.0101 g. Dry matter content (g) was used to calculate biomass allotment, following a study by Kaur et al. (2019).

The impacts of *S. marianum* population on communities’ diversity were measured by using species richness (S), Shannon–Wiener diversity index (_H’_), and species evenness index (E) in the field during evaluation of phytosociological attributes and biomass assessment.


(1) $$H^{\prime }=-\sum_{i=1}^{S}p\,i\,\text{ln}\,p\,i$$

(2) $$\text{E}=\frac{H^{\prime }}{\text{InS}}$$

Where *pi* = proportion of the species (*i*) to total number of species, i.e., n/N (number of individual/total number of individuals) and In = natural logarithm.

### Soil Analysis and Biotic Factor Scaling

Soil samples were collected from the center of each quadrate by digging to a 5–15 cm depth. These samples were then mixed carefully to produce replicates (*n* = 3). The soil had been air-dried, screened, and analyzed for textural classes using the hydrometric technique of a study by Huluka and Miller (2014). The Kjeldahl procedure was adopted to determine total nitrogen (N) (%), while the ammonium vanadate molybdate method was used to determine total phosphorus (P) (mg/kg). Similarly, total potassium (K) (mg/kg) was estimated using the ammonium acetate extraction method and organic carbon (OC) (%) was determined using the protocol by Tandon (1993). A digital conductometer (Model CC601 Century) was calibrated using a 0.01 M KCl solution to determine the soil sample’s electrical conductivity (EC) (S/cm) following Tandon’s technique (1993). Aspect angle was measured using a clinometer and GPS latitude and longitude were recorded for each elevation zone.

A six-point scale (0–5) was developed to measure the degrees of biotic disturbance caused by erosion, agricultural field’s disturbance, grazing, and traffic density. A plot with a score of 0 was considered undisturbed, while one with a score of 5 was considered extremely disturbed (Mligo, 2011). Thus, 0 represents no disturbance, 1 represents 0–20% of the plot disrupted, 2 represents 21–40% of the plot disturbed, 3 represents 41–60% of the plot disturbed, 4 represents 61–80% of the plot disturbed, and 5 represents 81–100% of the plot disturbed. This was a semi-quantitative evaluation and the degrees of disturbance were scaled based on the percentage of the given parameter persisting in a disturbing plot of 10 m × 10 m, with each kind of disturbance being examined separately. To account for different types of biotic disturbance, the point scale values were calculated following a study by Barry (2006) and Liu and Watanabe (2013).

### Statistical Analyses

The effects of invasion on PFTs were assessed using descriptive statistics and ANOVA. Significant effects were determined using *post-hoc* Tukey’s honestly significant difference (HSD) test for multiple comparisons of group means, with significance set at *p* < 0.05. The correlation functions and generalized linear model were used to analyze the relationship of elevation with PFTs to quantify the predictor strength. The SPSS version 22 was used to analyze all the data and Sigma plot version 14 provided a graphical representation. Cluster analysis was used to elucidate the PFTs grouping based on elevation, while redundancy analysis (RDA) was utilized to evaluate the relationship of traits with environmental, diversity, and soil variables. RDA was preferred, as its results and accuracy were more reliable than other ordination techniques.

## Results

### Morphological Traits and Biomass Allocation

The morphological traits and biomass allocation changes were significant across the elevation gradient in conjunction with IVI. Most of the traits and biomass, i.e., AGPH/P, WS, DWS/P, NL/P, LL/L, LW/L, DWL/P, NF/P, NS/P, CC/P, VDW/P, and TDW/P, show regular decrease with the decrease of *S. marianum* IVI from groups I-III (Table 1). The highest decrease in the traits was observed for CC/P (40%), followed by DWS/P and AGPH/P (27% each), while the lowest decrease was observed for DWL/P (5%). In contrast, a few traits, i.e., LR, WR, and DWR/P, show irregular variation, i.e., decrease from group I to group II and then increases where the total increases recorded for these traits were 37.4, 27.38, and 46.35%, respectively.

**TABLE 1:** Morphological and biomass traits variations represented as mean and SE with ANOVA output of *Silybum marianum* (*S. marianum*) along the elevation gradient in Pakistan.

Trait	Group I	Group II	Group III	*F*-value	*P*-value
IVI	100 ± 0*^a^*	63 ± 7*^b^*	33 ± 6*^c^*	560	2.13E-28
RL	4.55 ± 0.10^a^	3.86 ± 0.14^a^	6.17 ± 0.16^b^	5.27	0.0096
RD	2.31 ± 0.05^a^	2.28 ± 0.06^a^	3.14 ± 0.04^b^	6.36	0.0042
DWR/P	3.1 ± 0.07^a^	2.94 ± 0.08^a^	5.48 ± 0.16^b^	10.70	0.00021
AGPH/P	131.98 ± 0.85^a^	112.39 ± 0.57^b^	95.99 ± 0.59^c^	52.92	1.4E-11
SD	5.22 ± 0.05^a^	4.91 ± 0^a^	4.36 ± 0.02^b^	10.56	0.00023
SDW/P	32.49 ± 0.2^a^	27.60 ± 0.14^b^	23.49 ± 0.14^c^	52.56	1.54E-11
NL/P	27.19 ± 0.10^a^	26.48 ± 0.08^b^	23.93 ± 0.09^b^	24.76	1.49E-07
LL/L	35.19 ± 0.10^a^	34.49 ± 0.08^b^	31.93 ± 0.09^b^	24.81	1.46E-07
LW/L	16.60 ± 0.05^a^	16.24 ± 0.04^b^	14.96 ± 0.04^b^	24.81	1.46E-07
LDW/P	22.59 ± 0.05^a^	22.24 ± 0.04^b^	20.96 ± 0.04^b^	24.81	1.46E-07
NC/P	22.97 ± 0.25^a^	22.48 ± 0.11^b^	19.19 ± 0.11^b^	9.45	0.0005
NS/P	91.90 ± 1^a^	89.91 ± 0.45^b^	76.79 ± 0.48^b^	11.07	0.00017
CC/P	79.98 ± 0.85^a^	60.22 ± 0.57^b^	43.14 ± 0.50^c^	60.28	2.28E-12
VDW/P	58.19 ± 0.34^a^	52.77 ± 0.26^b^	49.94 ± 0.36^b^	24.03	2.05E-07
RDW/P	2.72 ± 0.09^a^	2.79 ± 0.01^a^	2.76 ± 0.007^a^	0.9946	0.3796
TDW/P	60.9 ± 0.24^a^	55.54 ± 0.17^b^	52.70 ± 0.26^b^	24.0833	2.0E-07

*IVI, Importance value index; RL, Root length, cm; RD, Root diameter, cm; DWR/P, Dry weight root/Plant, g; AGPH/P, Above ground plant height/Plant, cm; SD, Stem diameter, cm; SDW/P, Stem dry weight/Plant, g; NL/P, Number of leaves/Plant; LL/L, Leaf length/L, cm; LW/L, Leaf width/Leaf, cm; LDW/P, Leaf dry weight/Plant, g; NF/P, Number of Capitula/Plant; NS/P, Number of seeds/Plant; CC/P, Crown cover/Plant, cm; VDW/P, Total dry weight/Plant, g; RDW/P, Reproductive dry weight/Plant, g; TDW/P, Total dry weight/Plant, g. Different superscript indicate significant difference at p < 0.05.*

**FIGURE 3:**
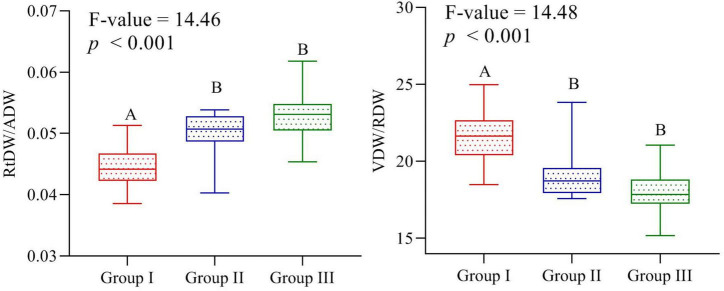
Comparison of root dry weight/aboveground dry weight and vegetative dry weight/reproductive dry weight ratios among the three groups separated on an elevation basis. Different letters indicate significant difference at *p* < 0.05.

**FIGURE 4:**
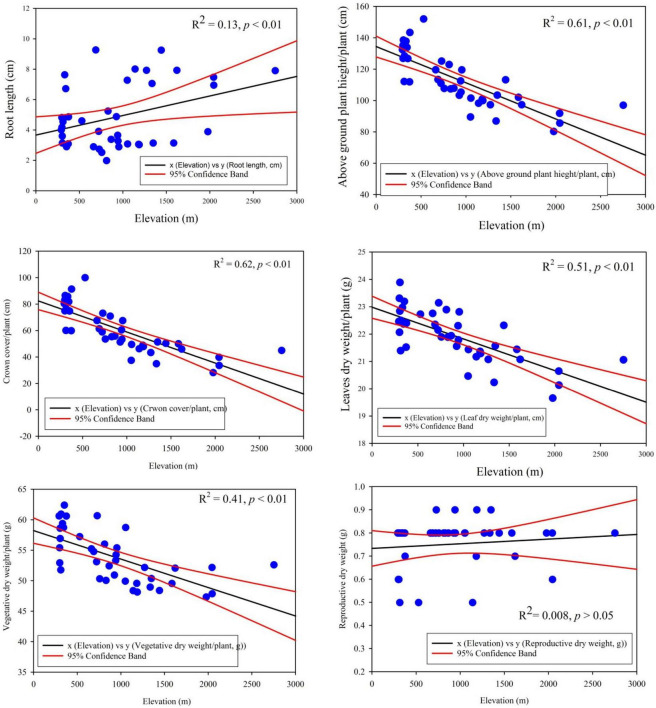
Linear regression model outputs of important morphological and biomass traits across the elevation gradients in *Silybum marianum*.

The linear regression model displayed significant relationship of traits plasticity in response to the elevation gradient, such as CC/P (*R*^2^ = 0.62, *p* < 0.01), AGPH/P (*R*^2^ = 0.61, *p* < 0.01), LDW/P (*R*^2^ = 0.51, *p* < 0.01), VDW/P (*R*^2^ = 0.41, *p* < 0.01), and RL/P (*R*^2^ = 0.13, *p* < 0.01) (Figure 3). In contrast, reproductive dry weight, i.e., RDW/P (*R*^2^ = 0.008, *p* > 0.05) exposed noteworthy non-significant variation across the elevation (Figure 4). Overall, the PFTs of *S. marianum* presented an imperative relationship with elevation.

### Invasion Effects on Related Soil and Diversity Variables

In soil and diversity characteristics, OM (%) (*F* = 4.46, *p* < 0.01), pH (*F* = 4.47, *p* < 0.01), lime (%) (*F* = 3.41, *p* < 0.05), S (*F* = 130, *p* < 0.0001), H (*F* = 348, *p* < 0.001), and J (*F* = 1,631, *p* < 0.0001) presenting significant changes with elevation coupled with fluctuations in *S. marianum* IVI; however, soil texture and hydraulic properties varied non-significantly with the increasing elevation (Table 2). The effect of *S. marianum* IVI was very much pronounced on the diversity of species compared to the soil variables. Both the soil and diversity indices were correlated with variations in IVI. The IVI exhibited a strong positive correlation with pH, OM (%), FC, and AW. Diversity indices and elevation revealed a strong negative correlation and N (%), P (mg/kg), and soil texture showed a non-significant correlation (Table 1). Soil nutrients such as OM (%) and N (%) were maximum at low elevation and minimum at high elevation (Table 3). The diversity indices show increasing trends with a decrease in the IVI of *S. marianum*. Group I has the lowest diversity indices, while group III has the highest diversity indices. The species evenness index varies from extreme 0 to 0.9 ± 0.003 among the groups.

**TABLE 2:** Soil and diversity parameters changes with *S. marianum* invasion along the elevation gradient in Pakistan.

Variable	Group I	Group II	Group III	*F*-value	*P*-value
CL %	29.97 ± 0.6	33.18 ± 0.69	35.17 ± 0.71	1.13	0.33
SL %	38.02 ± 0.68	32.81 ± 0.60	33.68 ± 0.66	1.33	0.28
SN %	32.07 ± 0.74	34.01 ± 0.73	31.15 ± 0.43	0.39	0.68
pH	6.21 ± 0.06^a^	6.25 ± 0.60^b^	5.55 ± 0.03^b^	4.47	0.01
OM%	0.81 ± 0.03^a^	0.58 ± 0.02^a^	0.49 ± 0.005^b^	4.46	0.01
Lim %	10.07 ± 0.16^a^	10.13 ± 0.22^a^	12.58 ± 0.23^b^	3.41	0.04
N %	6.68 ± 1.17	4.699923077	0.37 ± 0.01	0.91	0.41
EC	33.15 ± 0.93	42.92 ± 0.82	40.5 ± 0.72	2.79	0.07
K	142.69 ± 2.85	133 ± 2.27	153.57 ± 2.96	1.07	0.35
WP	0.19 ± 0.003^a^	0.19 ± 0.003^a^	0.15 ± 0.002^b^	3.91	0.02
FC	0.33 ± 0.003^a^	0.33 ± 0.003^a^	0.29 ± 0.002^b^	4.64	0.01
BD	1.30 ± 0.008^a^	1.32 ± 0.007^a^	1.23 ± 0.005^b^	3.53	0.04
SP	0.50 ± 0.003^a^	0.51 ± 0.003^a^	0.47 ± 0.002^b^	3.63	0.03
AW	0.14 ± 0.001^a^	0.14 ± 0.001^a^	0.13 ± 0.0007^b^	4.46	0.01
S	1 ± 0^a^	6.15 ± 0.11^b^	9.29 ± 0.13^c^	130.4	1.11E-16
H	0^a^	1.45 ± 0.02^b^	1.99 ± 0.01^c^	348.8	1.11E-16
J	0^a^	0.81 ± 0.004^b^	0.9 ± 0.003^c^	1631	1.11E-16

*Lat, Latitude; Long, Longitude; Elev, Elevation; AD, Aspect degree; CL%, Clay percentage; SL%, Silt percentage; SN%, Sand percentage; pH, Protenz Hydrogen; OM%, Organic matter percentage; Lim%, Lime percentage; N%, Nitrogen percentage; EC, Electrical conductivity; K, Potassium, mg/kg; WP, Wilting point; FC, Field capacity; BD, Bulk density; SP, Saturation point; S, Species richness; H, Shannon-wiener diversity index; J, Pielou evenness index. Different superscript indicate significant difference at p < 0.05.*

**TABLE 3:** Variation of spatial parameters represented as mean and SE along the elevation gradient in Pakistan.

Variable	Group I	Group II	Group III	*F*-value	*P*-value
Lat. (°)	34.62 ± 0.03^a^	34.36 ± 0.02^a^	34.82 ± 0.01^b^	6.37	0.004
Long. (°)	71.90 ± 0.02	72.07 ± 0.02	72.04 ± 0.02	1.25	0.299
Elev.	340.15 ± 4.84^a^	859.38 ± 13.75^b^	1548.64 ± 35.37^c^	50.81	0.000
AD	93.15 ± 3.18	95.54 ± 3.57	118.5 ± 3.25	1.35	0.27
EI	3.08 ± 0.3^a^	9.38 ± 0.44^a^	30.21 ± 0.62^b^	62.17	1.47E-12
CF	50.41 ± 1.18^a^	33.31 ± 0.61^b^	18.43 ± 0.78^c^	22.19	4.64E-07
GP	2.91 ± 0.24^a^	15.38 ± 0.55^b^	35.36 ± 0.78^c^	57.52	4.42E-12
TD	75.41 ± 0.91^a^	44.61 ± 0.53^b^	24.28 ± 0.33^c^	22.47	1.11E-16

*Lat, Latitude, °; Long, Longitude, °; Elev, Elevation, m; AD, Aspect degree, °; E, Erosion intensity; C, Cultivated fields; G, Grazing pressure; TD, Traffic density. Different superscript indicate significant difference at p < 0.05.*

### Associated Spatial and Biotic Variables of *Silybum marianum* Distributed Sites

Similarly, in geographic variables, latitude (*F* = 6.37, *p* < 0.05) and elevation (*F* = 50, *p* < 0.01) significantly vary with changes in the IVI. The highest variation was found for elevation, indicating that it is a prominent factor in effecting the morphological traits and biomass allocation. Longitude and aspect degree show insignificant variation across the site invaded. Moreover, the biotic factors vary across the elevation and IVI gradients, i.e., traffic density and cultivated fields percentage, were higher at lower altitudes, increased at higher altitudes, and varied significantly (*F* = 22, *p* < 0.001). However, the reverse was true for grazing and erosion percentages and varied significantly, having *F* = 57.53 and 62.17, respectively (*p* < 0.001).

### Relationship of Traits With Environmental Variables

In RDA, axis 1 shows 62.6% of variance accommodating the major bulk and explained significant characteristics related to species traits and environment. The total variance on the three axes is 78.9% revealing little bulk of factors on axes 2 and 3. Similar trends were also observed regarding the distribution of Eigen vectors on the ordination axes. Moreover, possible combination, i.e., Monte Carlo, showed that the relationship between the characteristics and the environmental variables chosen by the model was meaningful. The response-prediction model revealed by coefficient correlation is also significant having *r*-value of 0.96, 0.93, and 0.92 on axes 1, 2, and 3, respectively.

**FIGURE 5:**
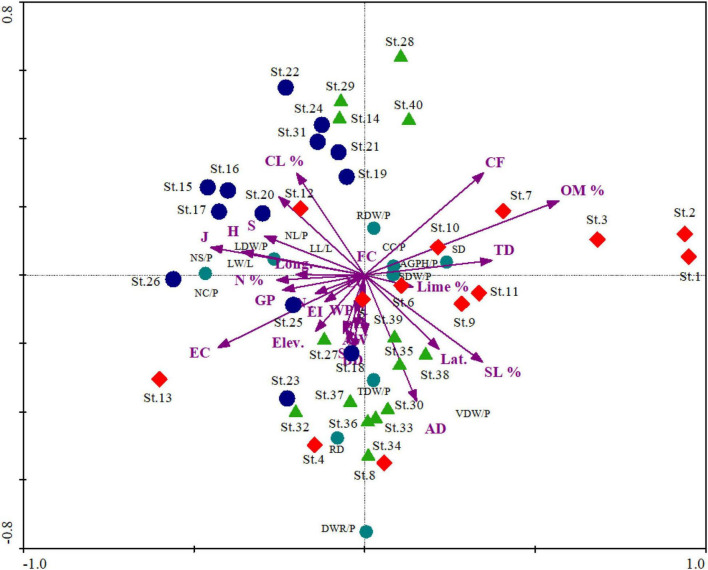
Redundancy analysis biplot showing the distribution pattern of sites and different morphological and biomass traits. St (Site number): The rest of the captions are the same as that mentioned in Tables 1–3.

The first axis was defined negatively by some of the environmental variables such as elevation (*r* = −0.79, *p* < 0.01), lime (%) (*r* = −0.46, *p* < 0.01), S (*r* = −0.82, *p* < 0.01), H (*r* = −0.81, *p* < 0.01), J (*r* = −0.73, *p* < 0.01), EI (*r* = −0.73, *p* < 0.01), and GP (*r* = −0.74, *p* < 0.01), as given in Table 4. The diversity indices, i.e., species richness, Shannon–Wiener diversity index, evenness index, grazing, and erosion intensity, show a negative relationship in the ordination axes with the importance value index revealing homogenization of the communities. In contrast, certain variables such as IVI (%) (*r* = 0.84, *p* < 0.01), pH (*r* = 0.32, *p* < 0.05), FC (*r* = 0.32, *p* < 0.05), AW (*r* = 0.31, *p* < 0.05), CF (*r* = 0.62, *p* < 0.01), and TD (*r* = 0.76, *p* < 0.01) exhibit strong positive correlation on axis 1. In the biplot scores, the importance value index shows positive highest score on axis 1, while diversity indices and elevation show negative scores that further confirm the negative relationship of diversity with the importance value index. The RDA variables depicted in biplot revealing the significant factors affecting the biomass allocation across the importance value gradient (Figure 5).

**TABLE 4:** Correlation and biplot scores extracted from redundancy analysis of the associated environmental variables operated on *S. marianum* plant functional traits (PFTs).

Variable	Correlation			Biplot scores		
	Axis 1	Axis 2	Axis 3	Axis 1	Axis 2	Axis 3
1 IVI	0.849	0.073	–0.336	2.558	0.094	–0.28
2 Lat	–0.237	–0.416	0.107	–0.714	–0.538	0.089
3 Long	–0.025	–0.156	0.327	–0.074	–0.201	0.272
4 Elev	–0.794	–0.16	0.244	–2.393	–0.207	0.203
5 AD	–0.209	–0.267	–0.115	–0.628	–0.345	–0.096
6 CL%	–0.269	0.131	0.327	–0.81	0.169	0.272
7 SL%	0.052	–0.2	–0.412	0.157	–0.259	–0.343
8 SN%	0.236	0.068	0.078	0.712	0.088	0.065
9 pH	0.328	–0.052	–0.352	0.988	–0.067	–0.293
10 OM%	0.14	0.356	–0.608	0.421	0.461	–0.506
11 Lim%	–0.466	–0.054	–0.031	–1.403	–0.07	–0.025
12 N%	0.265	–0.012	0.045	0.8	–0.015	0.037
13 EC	–0.064	0.066	0.236	–0.193	0.086	0.196
14 K	–0.172	–0.063	0.19	–0.518	–0.081	0.158
15 WP	0.279	–0.115	–0.307	0.84	–0.149	–0.256
16 FC	0.321	–0.12	–0.373	0.967	–0.155	–0.31
17 BD	0.258	–0.129	–0.366	0.778	–0.167	–0.304
18 SP	0.305	–0.111	–0.349	0.918	–0.144	–0.291
19 EC	0.231	0.094	0.11	0.695	0.122	0.091
20 AW	0.315	–0.097	–0.351	0.95	–0.125	–0.292
21 S	–0.818	0.096	0.34	–2.464	0.125	0.283
22 H	–0.81	0.072	0.366	–2.439	0.093	0.305
23 J	–0.726	0.104	0.411	–2.187	0.134	0.342
24 EI	–0.735	–0.092	0.285	–2.215	–0.119	0.237
25 CF	0.607	0.337	–0.453	1.83	0.435	–0.377
26 GP	–0.737	–0.019	0.318	–2.221	–0.025	0.265
27 TD	0.758	0.082	–0.502	2.282	0.107	–0.418

*Captions are the same as that of Tables 2, 3.*

## Discussion

High elevations have a greater diurnal variation in climatic conditions (Pandey et al., 2006). In *S. marianum*, this study found evident and substantial differences in PFTs over the altitudinal gradient. Ullah et al. (2022) in *Xanthium strumarium* (*X. strumarium*) and Rathee et al. (2021) in *Parthenium hysterophorus* (*P. hysterophorus*) found similar trends of RL/P, AGPH/P, and RDW characteristics, while Hattori et al. (2016) in *Impatiens textori* Miq. found similar pattern variations in flower dimension, RL/P, and AGPH/P. Similarly, in *Ferula jaeschkeana*, Vatke’s growth dynamics were reported by Yaqoob and Nawchoo (2017), while Datta et al. (2017) disclosed *Ageratina adenophora* (Spreng.) R. M. King and H. Rob life cycle phases. AGPH/P, which may protect plants from strong winds and cold stress, remains the preeminent noteworthy alteration in vegetative features (Fabbro and Körner, 2004). It is possible that the closer a plant stands to the ground’s surface, the more likely it is to successfully produce seeds and distribute them (Fabbro and Körner, 2004). Trunschke and Stöcklin (2017) discovered that reducing height might maintain reproductive fitness. Yaqoob and Nawchoo (2017) found that RDW increased with elevation, consistent with our results. Due to varied topography, individual mountain ranges may have links with environmental fundamentals and elevation (Olejniczak et al., 2018).

Elevation affects soil characteristics (Badía et al., 2016); for example, with the rise in altitude, the percentage OM, percentage N, and EC decrease, while the percent lime and K (mg/kg) increase. Sardans et al. (2017) reported that soil nutrient availability and chemical composition are either directly or indirectly governed by a plant species’ potential to invade. In addition, Vasquez et al. (2008) reported that invasive species can use available soil nutrients that determine their growth progression or decline. Alien invasive plants may also contribute to soil nutrient homogeneity and, thus, stimulate further incursions in populated regions (Dassonville et al., 2008). Carbon, nitrogen, phosphorus, and potassium are elevated in invaded sites (Sardans et al., 2017). According to a study by Cowie et al. (2019), plant growth rate and biomass production increased under nutrient-rich conditions (along with changes in plant biomass), which are in line with our findings. Evidence suggests that invaders such as *P. hysterophorus* disruption (Seta et al., 2013; Rathee et al., 2021) and *X. strumarium* (Ullah et al., 2021) have been shown to benefit from environmental degradation. The combination of soil instabilities and fertilizers inputs are the most critical factor in promoting and developing non-native species (Hobbs and Huenneke, 1992). According to studies, plant invasion and soil nutrition have a substantial relationship (Hester and Hobbs, 1992). Osunkoya et al. (2017) found increased breakdown and microbiological activity in soils infested by *P. hysterophorus*. In this situation, where nutrient-rich regions are mostly invaded, the same has been true for *S. marianum*. Our findings suggest that reduced nutrient availability at higher elevations over 1,000 m may diminish biomass allocation to aboveground parts, especially vegetative organs.

Even though most native species are threatened by climate change, aliens that thrive in warm areas are expected to prosper (Hou et al., 2014). Global elements such as rainfall, nitrogen, and carbon dioxide deposition are also connected to plant invasion and development (Hou et al., 2014). According to various scientists, increased precipitation boosts nutritional richness and invasive species (Eskelinen and Harrison, 2014). The results of this study show that *S. marianum* is capable of adapting to a broad range of environmental gradients. Its robust growth and multiplication show its ability to thrive in a wide range of climate and soil conditions. Several studies reveal that invasive species such as *P. hysterophorus* exhibits comparable phenotypic flexibility and a broad spectrum of environmental adaptation (Datta et al., 2017; Khan et al., 2020) and the same is true for *S. marianum*.

According to a study by Annapurna and Singh (2003) and Kadam et al. (2009), varied soil types and levels of environmental variables had a pronounced effect on the species plasticity. Invasive plants are well adapted to changes in climate variables such as CO_2_, water scarcity, and higher temperatures (Nguyen et al., 2017). Allometric variations indicate that how plants allocate biomass in natural environments and resources adaptability methods (Weiner, 2004). The allocation of resources, reproduction, and stress tolerance were probably the most important mechanisms that modify species fitness (Fabbro and Körner, 2004). Resource allocation and trade-offs vary across populations and individuals in response to biotic and abiotic environments (Redmond et al., 2019). Similarly, *S. marianum* changed its traits and biomass allocation according to habitat types and conditions along the elevation gradients.

*Silybum marianum* PFTs output increase at lower elevations because plants can easily take nutrients from nutrient-rich soils at lower altitudes (Berntson and Wayne, 2000). These findings were in line with Datta et al. (2017), who found that *Ageratina adenophora* biomass increased at lower altitudes. In contrast, Zhigang et al. (2006) revealed that higher altitudes have higher nutrient concentration and might be responsible for the observed tendency in *Anemone rivularis* Buch.–Ham. ex DC. and *Anemone obtusiloba* D. Don biomass allocation that do not decrease with elevation. Invading species need more reproductive biomass than vegetative biomass to complete their life cycles when confronted with environmental stresses such as temperature, nutrients, and uptake light intensity along with an elevational gradient (Wenk and Falster, 2015). A similar adaption strategy was discovered by Arroyo et al. (2013) in mountain habitats, where plants maintained reproductive output even at higher elevations (2013). We found that when elevation increases, *S. marianum*’s reproductive ability takes priority over its vegetative ability. Moreover, soil OM and N%, EC, N, and longitude may also have a role in PFT plasticity; this has been shown by researchers such as Mason et al. (2017); Xue et al. (2018), and Chen et al. (2020).

Biotic factors significantly contributed to disturbing native community structure, favoring the invasion of non-native species (Geiger and McPherson, 2005). This study assessed erosion intensity, grazing pressure, cultivated fields, and traffic density to relate the specie invasion and biotic factors. These factors vary significantly across the IVI and elevation gradients, indicating that they are linked with species invasion and community disturbance. Traffic density and agricultural activities or areas occupied by cultivated fields were higher at lower altitudes, making the communities more prone to invasion and this may be due to the easy transportation of the invasive plant’s propagules in such areas, as reported by Pretto et al. (2010); Khan et al. (2020), and Chhogyel et al. (2021). In contrast, grazing pressure and erosion intensity increased at higher altitudes that favor invasion by providing empty niches for newly invading propagules and, thus, enabling the plant to invade the higher altitudes areas, as reported by McDougall et al. (2005) and Sjödin et al. (2008).

The plant’s ability to adapt to climatic change across altitude gradients may be aided by genetic divergence, genetic drift, and phenotypic adaptability (Gonzalo Turpin and Hazard, 2009). Vasseur et al. (2018) revealed the importance of a specific gene in *Arabidopsis thaliana* and reported that these genes respond to abiotic stress and local temperature that affect growth allometry. On the other hand, the species adopted various methods to allocate their biomass at various altitudes. As a result, we observed *S. marianum* phenotypic plasticity and vegetative and reproductive biomasses were intimately correlated to elevation. These had a good relationship with L%, OM%, N%, and environmental variables. Similar patterns of environmental factors influencing the allocation of biomass and plasticity in two *Gentiana* species along an altitude gradient were found in China’s Yunnan-Guizhou Plateau by Zhang et al. (2020). Examining plasticity approaches across environmental gradients further to better understand that additional environmental variables and genetic components are necessary.

## Conclusion

Lowland regions have been considered as a hotspot for invasion; however, due to human interference, the process continues to higher elevations (direct or indirect), indicating that mountain ecosystems are no longer immune to invasion. This study implies that *S. marianum* alters its traits to expand its habitat and geographic range to higher elevation. The results found that *S. marianum* potential to adapt in the environmental condition across the elevation is dependent on its ability to reproduce. This study also finds that the diversity indices of the communities are related to the invasive species phytosociological attributes, i.e., importance value index. Therefore, *S. marianum* and other invasive plant species with comparable phylogenetic or morphological characteristics may be anticipated by this approach. Study on the spread and development of foreign invasive species over climatic gradients is critical to our understanding and predicting the effects of climate change on these species. To the best of our knowledge, this is the first ever study that investigates the invasiveness of *S. marianum* across the elevation gradient by studying its PFTs and will open a new insight into its ecology and invasiveness in the other regions as well. Moreover, biotic interaction of the plant species and anthropogenic factors may be considered to better understand the future invasion perspective of the species for management and conservation of the native communities.

## Data Availability Statement

The original contributions presented in this study are included in the article/supplementary material, further inquiries can be directed to the corresponding author.

## Author Contributions

NK, RU, MA-M, and HA planned and designed the research. NK, RU, SA, YA, AA-H, MA-M, MO, and HA performed the experiments and analyzed the data. SA, YA, AA-H, MA-M, and MO contributed to the reagents/chemicals. NK and HA provided a draft version of the manuscript. All authors revised and finalized the manuscript.

## Conflict of Interest

The authors declare that the research was conducted in the absence of any commercial or financial relationships that could be construed as a potential conflict of interest.

## Publisher’s Note

All claims expressed in this article are solely those of the authors and do not necessarily represent those of their affiliated organizations, or those of the publisher, the editors and the reviewers. Any product that may be evaluated in this article, or claim that may be made by its manufacturer, is not guaranteed or endorsed by the publisher.
